# Reviewing the *Clostridioides difficile* Mouse Model: Insights into Infection Mechanisms

**DOI:** 10.3390/microorganisms12020273

**Published:** 2024-01-27

**Authors:** José L. Fachi, Marco A. R. Vinolo, Marco Colonna

**Affiliations:** 1 Department of Pathology and Immunology, Washington University School of Medicine, St. Louis, MO 63110, USA; mcolonna@wustl.edu; 2 Department of Genetics and Evolution, Microbiology and Immunology, Institute of Biology, University of Campinas, Campinas 13083-862, SP, Brazil; mvinolo@unicamp.br

**Keywords:** *Clostridioides difficile*, gut, colitis, innate immunity, intestinal epithelial cells

## Abstract

*Clostridioides difficile* is an anaerobic, spore-forming bacterium associated with intestinal infection, manifesting a broad spectrum of gastrointestinal symptoms, ranging from mild diarrhea to severe colitis. A primary risk factor for the development of *C. difficile* infection (CDI) is antibiotic exposure. Elderly and immunocompromised individuals are particularly vulnerable to CDI. A pivotal aspect for comprehending the complexities of this infection relies on the utilization of experimental models that mimic human CDI transmission, pathogenesis, and progression. These models offer invaluable insights into host–pathogen interactions and disease dynamics, and serve as essential tools for testing potential therapeutic approaches. In this review, we examine the animal model for CDI and delineate the stages of infection, with a specific focus on mice. Our objective is to offer an updated description of experimental models employed in the study of CDI, emphasizing both their strengths and limitations.

## 1. Introduction

*Clostridioides difficile*, commonly referred to *as C. difficile* or *C. diff*, is a spore-forming anaerobic bacterium of significant notoriety within the domain of infectious diseases [1]. Over recent decades, it has emerged as a prominent causative agent of healthcare-associated infections, particularly affecting individuals who have recently undergone antibiotic therapy or present a compromised immune system [2]. The infection primarily relies on the perturbation of the gut microbiota [3], creating a conducive environment for the colonization of *C. difficile* and the subsequent release of its toxins [4]. These toxins adversely affect host cells, undermining the integrity of the intestinal barrier and causing inflammation along with tissue damage [5,6]. The clinical manifestation of the disease in humans encompasses a spectrum of presentations, ranging from mild diarrhea to severe colitis, often leading to life-threatening complications and, in some cases, mortality [7,8].

Substantial research efforts have been dedicated to unraveling the complexities of *C. difficile* infection (CDI), covering aspects such as prevention, pathogenesis, and treatment approaches. An essential component of this research journey involves the establishment and utilization of animal models to replicate and analyze various aspects of diseases. For decades, hamsters and mice have been the primary animal models for studying CDI [9]. These models have been instrumental in elucidating key aspects of the disease, such as *C. difficile* toxins production, host immune response, and the role of microbiota in the host susceptibility to CDI. However, these models inherently exhibit limitations that should be taken into consideration for experimental applications.

In this review, we offer updated information on animal models in *C. difficile* infection research, with a particular focus on murine models. We aim to emphasize the valuable insights these models have brought to our comprehension of CDI and highlight their effective performance in elucidating the disease’s complexity.

## 2. *Clostridioides difficile* Infection

“*Bacillus difficile*”, as denominated before, entered the scientific spotlight in 1935 when it was first identified by researchers Hall and O’Toole [10]. At that time, this bacterium was considered relatively innocuous, quietly inhabiting the human gut without drawing much attention. However, it was during the 1970s that “*Clostridium difficile”* began to reveal its pathogenic potential [11,12,13]. Researchers observed a connection between *C. difficile* and antibiotic-associated diarrhea, as well as pseudomembranous colitis, leading to a surge in research interest [8]. Since then, the bacterium has persisted in its evolutionary trajectory, developing resistance to various antibiotics and producing virulent strains that pose significant challenges to healthcare providers and researchers alike [14]. In 2016, the reclassification of “*Clostridium difficile*” to “*Clostridioides difficile*” marked a significant taxonomic shift, motivated by advancements in genetic research and DNA sequencing [15]. This realignment sought to more accurately reflect the bacterium’s genetic and evolutionary characteristics, setting it apart from the *Clostridium* genus.

*C. difficile* is a Gram-positive bacterium characterized by its ability to form spores [16]. These spores are the resilient, dormant form of the bacterium, exhibiting the capacity to endure adverse conditions, including high temperatures and sterilizing chemicals [17]. Their durability contributes to their persistence in the environment, making CDI prevention and control challenging. Moreover, *C. difficile* spores play a critical role in CDI recurrence; even after successful CDI treatment, some patients experience a recurrence due to the germination of these spores when favorable conditions arise in the colon, often as a result of disruptions to the normal gut microbiota [16,18,19]. Certainly, their germination and the onset of *C. difficile* infection depend not only on microbiota imbalance, but also on various host-related factors, including immunity, age, and others. In fact, individuals within healthcare settings face an increased risk for CDI, attributed to factors like the use of antibiotics, an impaired immune system, and direct exposure to an environment with a higher abundance of *C. difficile* spores [4]. Moreover, 2.4% to 17.5% of healthy adults can asymptomatically carry *C. difficile*, serving as potential sources of transmission, both in healthcare environments and the community at large [20,21,22].

The pathological consequences of CDI are primarily centered in the colon. The disease can manifest across a spectrum of severity, ranging from mild diarrhea to severe pseudomembranous colitis, and in extreme cases, leading to fulminant outcomes [23,24,25]. Specifically, the development of pseudomembranous colitis arises as a sequence of unfolding events leading to colitis: CDI often starts with the ingestion of *C. difficile* spores, typically encountered in the environment, which then transform into active bacteria after germination within the large intestine (cecum and colon) [16]. *C. difficile* produces two crucial toxins, toxin A (TcdA) and toxin B (TcdB), that specifically target epithelial cells by binding to their respective receptors [5,6]. Once internalized, these toxins cause the subsequent inactivation of Rho-family GTPases through glucosylation [5,26,27]. This event disrupts the host cell’s cytoskeleton, accelerating cell death [28,29]. Consequently, the damaged epithelial layer increases gut permeability and facilitates the translocation of luminal content into the intestinal lamina propria. Simultaneously, the accumulation of dead cells and the presence of translocated bacteria enable immune cells and fluids to infiltrate the tissue [30,31]. As a result, pseudomembranes manifest as yellowish-white patches on the inflamed colon mucosa, consisting of inflammatory cells, mucus, fibrin, and dead epithelial cells [30]. The classic symptoms of pseudomembranous colitis include severe diarrhea, abdominal pain, and fever. In the absence of treatment or in cases of heightened host susceptibility to infection, severe complications may arise, including toxic megacolon, bowel perforation, sepsis, and, in some instances, death [32].

In addition, the emergence of hypervirulent strains, such as the NAP1/B1/027 variant, has emerged as a global public health concern by being associated with more severe and recurrent cases of CDI [33,34,35,36,37]. Specifically, the PCR ribotype 027 strain, commonly denoted as RT027, distinguishes itself with heightened virulence, characterized by elevated production of TcdA and TcdB toxins [38]. Infections with the RT027 strain are known for their high recurrence rates, impacting patient outcomes and placing a significant burden on healthcare systems. Remarkably, RT027 demonstrates resistance to certain antibiotics, notably fluoroquinolones, presenting challenges in treatment and contributing to persistent infections [39,40]. Furthermore, this strain is recognized for frequently producing the binary toxin, also known as CDT (*C. difficile* transferase), which is associated with increased severity and mortality in patients [41,42,43]. Unlike the more well-known toxins TcdA and TcdB, CDT functions as an ADP-ribosyltransferase, comprising two subunits, CDTa and CDTb. CDTa manages enzymatic activity, while CDTb facilitates entry into host cells [42]. Research indicates that this binary toxin plays a crucial role in enhancing the virulence of *C. difficile*, particularly through increased activation of the inflammasome via Toll-like receptor 2 (TLR2)-mediated priming [44]. This heightened inflammasome activation is implicated in the pathogenicity of *C. difficile* infection. Additionally, CDT-induced TLR2 activation leads to the release of proinflammatory cytokines and increased apoptosis of eosinophils, further contributing to the overall impact of CDT on the host immune response [45]. Comprehending the role of the binary toxin is crucial for unraveling host immune responses and developing novel therapeutic strategies for CDI.

The current strategy for managing and treating patients with CDI typically involves a multifaceted approach [46]. Treatment plans are customized to address the unique clinical condition of each individual, underscoring the crucial role of early diagnosis and intervention for effectively managing the disease. The first step is discontinuing the antibiotic that may have triggered the infection, if applicable, to halt the selective advantage *C. difficile* gains in an altered gut microbiome. The next crucial step is the administration of specific antibiotics, such as vancomycin or fidaxomicin, to target and eliminate *C. difficile* bacteria [46,47,48]. Metronidazole is designated as an alternative agent and has been omitted from the treatment of nonsevere CDI since the 2021 guidelines [49]. Additionally, supportive care, including adequate hydration and electrolyte management, plays a pivotal role in treatment, particularly for patients with severe diarrhea [46]. These strategies do not contribute to the restoration of the natural microbiota’s colonization, potentially explaining their modest effectiveness in terms of treatment outcomes and increased rates of relapse episodes [3]. Approximately 15–30% of patients who respond to antimicrobial therapy may experience recurrent CDI, and the risk escalates with each subsequent episode [50]. Studies indicate that older age and female sex are associated with an increased risk of recurrence [51]. It is also noteworthy to notice that recurrent CDI is not always attributed to the same strain, with a new strain being identified in 33–56% of recurrent episodes [52,53,54]. For the treatment of recurrent CDI, Bezlotoxumab (Zinplava), a monoclonal antibody targeting the toxin TcdB, helps prevent recurrences and mitigates damage to colonic epithelial cells [55].

Remarkably, fecal microbiota transplantation (FMT) surprisingly emerges as a highly potent therapeutic approach for treating recurrent or severe CDI [56,57]. This procedure entails the infusion of fecal matter from a healthy donor into the patient’s gastrointestinal tract, with the overarching goal of reestablishing a balanced gut microbiota. The transplanted microbiome, armed with its diverse arsenal of beneficial microbes, plays a crucial role in efficiently combatting the opportunistic *C. difficile* [3,58]. Meta-analyses examining the efficacy of FMT for recurrent, severe, or fulminant CDI consistently indicate promising outcomes [59,60]. FMT has demonstrated high success rates in preventing recurrent CDI, with significant reductions in recurrence compared to standard antibiotic therapy. Moreover, this therapeutic approach has shown effectiveness in treating severe and fulminant cases, contributing to improved clinical outcomes and reduced mortality rates. However, it is not without its drawbacks and potential risks [61]. One of the primary concerns is the lack of long-term safety data, as the consequences of introducing a new, diverse microbial community into a patient’s gut over time remain uncertain. FMT relies on donated fecal matter, which, despite rigorous donor screening and testing, may still carry undetected pathogens or infections that can be transmitted to the recipient [62,63]. There is also a risk of unintended alterations to the recipient’s microbiome, potentially leading to unexpected health issues [64,65]. Continuing research and ongoing debates focus on the application and long-term consequences of FMT, emphasizing the need for a thorough evaluation of its risks and benefits.

Overall, effectively combating CDI requires a comprehensive understanding of its microbiological foundations, insight into the underlying pathological processes, and the ability to navigate the complex epidemiological factors that contribute to its persistence and spread. Given these considerations, animal models of *C. difficile* infection play a pivotal role in providing insights into the pathogenesis, treatment, and prevention of CDI.

## 3. Animal Models of CDI

CDI has been studied using different animal species, including hamsters, guinea pigs, rabbits, mice, and rats, as reviewed before [9]. The hamster model is the most commonly used experimental model. On hamsters, CDI can be induced after ingestion of antibiotics, and colonization occurs through experimental challenge or environmental exposure to *C. difficile* [66,67,68]. The disease in this model primarily affects the cecum with some involvement of the ileum, resulting in diarrhea and fatal enterocolitis when exposed to toxigenic strains [69,70]. It is important to note that this model represents well the severe and lethal forms of the disease, but does not consistently display the full spectrum of CDI symptoms seen in humans [66,67,71]. The hamster model has been employed for three decades to investigate CDI therapy and disease mechanisms. Studies involving hamster vaccination have demonstrated that *C. difficile* toxoids (formalin-inactivated toxins) can offer protection against fatal outcomes in animal models [72].

Mice and rats are less susceptible to CDI than hamsters [73]. Nevertheless, murine models serve as valuable tools for studying CDI, given their genetic similarity to humans and enhanced translational relevance [74]. Mouse application allows researchers to control various factors such as genetics, environment, and diet, ensuring consistent experimental conditions. The availability of transgenic mice facilitates the examination of host factors and components of immune responses throughout the progression of the disease. There are well-established protocols for inducing *C. difficile* infection in mice, making them a standardized and widely adopted model in the field. Typically, laboratory mice are subjected to antibiotic treatment to disturb their gut microbiota, rendering them susceptible to the germination and colonization of *C. difficile*—mirroring the dynamics observed in humans [75,76]. Then, the mice are orally inoculated with *C. difficile* spores or vegetative cells. Over time, infected mice may develop symptoms similar to those seen in humans, such as diarrhea, weight loss, and other gastrointestinal issues [54,77]. The severity of symptoms varies depending on the *C. difficile* strain used and the susceptibility state of the mouse [78]. Researchers can monitor various parameters, including the progression of infection, changes in gut microbiota composition, histopathological alterations in the colon, and levels of *C. difficile* toxins in feces [79]. The mouse model is very useful for testing potential treatments and interventions, including new antibiotics, probiotics, dietary modifications, or vaccines designed to combat *C. difficile* infection.

Numerous mouse models have been created for studying the mechanisms behind *C. difficile* pathogenesis, and each has its own strengths for investigating different aspects of the disease [75]. The selection of a particular method is guided by the research goals, the specific strain of *C. difficile*, and the intended disease features. One common approach is to perform a single oral gavage with *C. difficile* spores, as it mimics the natural route of infection from contaminated surfaces [80,81]. To study recurrent infections, some models involve multiple rounds of challenge [50,54]. Alternatively, researchers can administer purified *C. difficile* toxins to study the role of toxins in disease pathogenesis [82,83,84,85]. Antibiotic pretreatment disrupts the gut microbiota, making mice more susceptible to colonization, reflecting conditions seen in patients with antibiotic-associated *C. difficile* disease [86,87,88]. Likewise, models designed to investigate colonization resistance explore the use of probiotics or microbiota components to enhance the mechanisms of resistance [88,89,90,91].

Gnotobiotic/germ-free mice can be colonized by *C. difficile* and exhibit intestinal pathology, primarily in the colon, with pseudomembrane formation similar to human disease [92,93,94,95]. This model in particular enables the exploration of microbiota or isolated groups of bacteria’s role in the development of CDI. Reeves et al. unveiled a significant delay in the onset of primary CDI and relapse in germ-free mice pretreated with probiotics [96]. Notably, the microbiome of mice undergoing moderate CDI and receiving probiotic treatment revealed a striking increase in the abundance of the *Lachnospiraceae* family during the initial CDI phase. Intriguingly, mice that were precolonized with the *Lachnospiraceae* isolate demonstrated a substantial decrease in *C. difficile* colonization, decreased levels of intestinal cytotoxin, and exhibited milder clinical symptoms and colonic histopathology. This effect was not observed in mice solely colonized with *E. coli* as a control. This study suggests novel therapeutic approaches for the treatment and prevention of CDI by utilizing bacterial species that potentially inhibit *C. difficile* growth. Likewise, a bacterial consortium named VE303 is currently under development as a potential treatment for high-risk CDI [97,98]. This oral treatment consists of eight nonpathogenic, nontoxigenic, commensal strains of *Clostridia* that have been shown to effectively restore a healthy gut microbial community, mitigate inflammation, and elevate levels of protective metabolites in mice [97,98]. However, the use of gnotobiotic mouse models has a much higher cost and is less practical when compared to conventional mice. Additionally, the absence of other microorganisms in these models makes them less representative of the human situation. Nonetheless, the utilization of germ-free mice is crucial for investigating the specific role of human microbiota in the development of CDI. Humanized mouse models, engrafted with human microbiota, provide an environment closer to human *C. difficile* infection [99,100,101,102]. These methods offer flexibility in investigating various aspects of *C. difficile* infection, from pathogenesis to potential interventions.

## 4. Antibiotic-Induced Murine Model of CDI

Antibiotic-induced murine models of CDI are specifically designed to mimic the clinical manifestations and pathological features of human CDI within a controlled laboratory setting [80,103]. Despite some limitations, this model has greatly contributed to our understanding of CDI and continues to be an essential component of CDI research. Antibiotics are employed to initiate gut dysbiosis, a crucial step in simulating the clinical conditions that lead to *C. difficile* expansion [87,104,105]. Commonly used antibiotics for this purpose include vancomycin, cefoperazone, and tetracycline, typically administered orally via drinking water supplementation [73,106,107,108,109]. Likewise, clindamycin is often linked to the development of CDI in humans, attributed to its effectiveness against a wide range of bacteria. It is administered systemically as an antibiotic, usually through intraperitoneal injection in mice. In essence, antibiotic treatment eliminates competing bacteria in the gastrointestinal tract, thereby creating an environment that favors the proliferation of *C. difficile* [110,111].

The selection of antibiotics varies according to the specific study goals and the desired level of gut dysbiosis, with the administration of these antibiotics for a predetermined period before introducing *C. difficile* to initiate infection. Giel et al. conducted a study on the antibiotic-induced mouse model of CDI to investigate *C. difficile* spore germination [18]. Their findings revealed that spores were more likely to germinate in antibiotic-treated mice, but this germination was mitigated by cholestyramine, a treatment that chelated bile salts. This observation led the authors to suggest that cecal bacterial populations in antibiotic-treated mice had a reduced capacity to modify taurocholate, a spore germinant factor, providing further support for the role of bile salts in *C. difficile* spore germination and infection development in the host [18]. Similarly, a ten-day antibiotic pretreatment with broad-spectrum cefoperazone in drinking water showed an increase in mice’s susceptibility to infection with *C. difficile* strain VPI 10463 [105]. This particular strain of *C. difficile* produces elevated levels of toxins, and experimental infection with it proves lethal in hamsters and, depending on the dose, in mice [80,112].

Studies on mouse models have extensively characterized infection with *C. difficile* spores. Lawley et al. [113] confirmed that a low number of spores (seven) of *C. difficile* per square centimeter of mucosal tissue constitutes the minimum infectious dose. In another study by the same authors, Lawley et al. explored spore transmission between healthy and immunocompromised mice, shaping infection control strategies [73]. This study demonstrated that virulent *C. difficile* can asymptomatically colonize the intestines of healthy mice, establishing a persistent carrier state. The authors convincingly illustrated that when *C. difficile* was shed in significant amounts, referred to as the “super-shedder” state, it promoted efficient transmission. In contrast, the carrier state, in which *C. difficile* shedding was lower, did not facilitate transmission to the same extent. In healthy mice, the infection induced mild mucosal inflammation. Conversely, transmission to mice with compromised innate immune responses (Myd88^−/−^) resulted in severe and frequently fatal intestinal disease, underscoring the role of host factors in CDI.

In some research scenarios, a combination of antibiotics may be utilized to induce a more profound disturbance in the gut microbiota, closely mirroring the clinical situation where patients often receive multiple antibiotics before developing CDI. Chen et al. [80] pioneered an innovative mouse model wherein C57Bl/6 mice were subjected to a 3-day oral exposure to a combination of kanamycin, gentamicin, colistin, metronidazole, and vancomycin, followed by an intraperitoneal administration of clindamycin 48 h later. Subsequently, the mice were challenged with varying doses of *C. difficile*. This mouse model faithfully replicated human *C. difficile* infections, inducing characteristic symptoms such as diarrhea, weight loss, and typical histological features. The severity of the disease in this mouse model was proportional to the challenge dose used, ranging from 2 × 10^2^ to 10^5^ CFU. In a parallel initiative, our research team modified the infection model, originally proposed by Chen et al., to incorporate a more aggressive dose of the vegetative form of *C. difficile* [79]. We found that treating 8-week-old male C57BL/6 mice with a combination of kanamycin, gentamicin, colistin, metronidazole, and vancomycin for 4 days, followed by clindamycin administration 24 h later, and subsequent infection in the next day with 10^8^ CFU of *C. difficile*, effectively induced weight loss, diarrhea, and intestinal inflammation.

Furthermore, researchers investigated the progression of *C. difficile* infection in correlation with the age of the mice [92]. The study utilized germ-free C57BL/6 mice aged 7 to 14 months, infected with the BI/NAP1 strain. Notably, older infected mice were critically ill within 48 to 72 h after infection, exhibiting noticeable cecitis and colitis upon gross and histological examination. These infected mice also displayed elevated levels of keratinocyte chemoattractant (KC, also known as Cxcl1), interleukin 1β, monocyte chemotactic protein 1 (MCP-1, also known as Ccl2), and granulocyte colony-stimulating factor (G-CSF). Conversely, they had decreased levels of interferon-γ, interleukin-12, and interleukin-10 when compared to the younger control group. This unique model not only sheds light on the role of the host response in disease development but also highlights its connection with the aging process. These findings are particularly significant due to the rising incidence and severity of *C. difficile* infection in older adults, particularly those aged 65 years and above, largely associated with the emergence of the BI/NAP1 strain [114,115].

## 5. CDI Pathogenesis and Disease Progression in Mice

**Figure 1:**
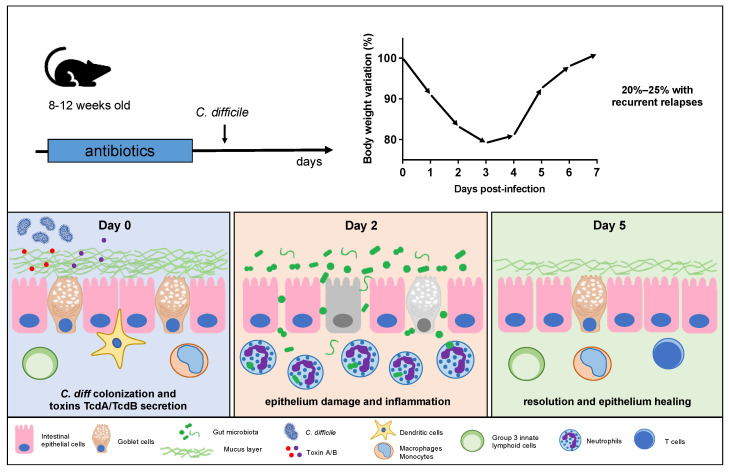
Pathogenesis of *C. difficile* infection in the mouse model. Mice are infected after antibiotic treatment. The disease is usually acute, lasting for less than a week for the first episode. After antibiotic treatment and microbiota disruption, *C. difficile* spores germinate and colonize the colon, releasing toxins that damage the intestinal epithelium. This damage can potentially lead to bacterial translocation and inflammation, primarily driven by neutrophil infiltration, which peaks around day 2 postinfection. On day 5 postinfection, a resolving phase begins, characterized by reduced inflammation and increased tissue repair mechanisms, including the involvement of ILC3 and Treg cells. Around 20–25% of infected mice may experience recurrent infection relapse, offering valuable insights into the role of adaptive immunity in CDI.

After antibiotic treatment, the mouse’s gut undergoes substantial modifications, rendering it susceptible to *C. difficile* colonization [87,116,117]. The highly resilient *C. difficile* spores colonize the intestines, primarily finding their niche in the murine cecum and colon [16,118]. Within these regions, spores undergo germination, transitioning into vegetative cells that actively grow and produce the toxins responsible for clinical symptoms (Figure 1) [118]. Toxin A (TcdA) and toxin B (TcdB) are released following *C. difficile* germination, typically occurring on the first day postinfection [118]. These toxins have a specific affinity for the intestinal epithelial cells, disrupting the colonic epithelium and subsequently contributing to tissue damage and bacterial translocation [119,120,121,122]. In particular, gut bacterial translocation involves the process through which bacteria, mostly those that naturally inhabit the intestinal tract, cross the protective barrier of the gut and enter the bloodstream or other distant anatomical regions. Furthermore, the compromised epithelial barrier plays a pivotal role in the development of diarrhea and inflammation during the early stages (around day 2 postinfection) [123]. This event can ultimately lead to the development of sepsis, a life-threatening condition that arises in an overwhelming and systemic inflammatory response [124]. The consequent uncontrolled immune reaction, often termed a “cytokine storm”, holds the potential to inflict substantial harm systemically, characterized by a spectrum of clinical symptoms, including a high fever, cognitive impairment, organ dysfunction, and a reduction in blood pressure [125,126]. In severe cases, mice may manifest fulminant colitis, elevating the risk of mortality significantly, while sepsis can lead to a fatal outcome [124].

In addition, the intestinal inflammatory cascade after TcdA and TcdB toxins-associated damage, which includes the recruitment of immune cells, such as neutrophils, and the release of proinflammatory cytokines, such as IL-1β, IL-23, TNF-α, and IFN-γ, occurs around 2 days postinfection, a hallmark of CDI disease progression [121,122,123,127,128,129,130]. In particular, these toxins have been noted for their ability to activate the inflammasome, a multiprotein complex that detects cellular stress and danger signals, instigating a potent inflammatory response [131]. This activation leads to the cleavage of proinflammatory cytokines, including interleukin-18 (IL-18) and IL-1β, crucial in combating the infection [132]. Excessive activation, however, can be pathogenic, contributing to heightened host morbidity and mortality [44,131]. Moreover, the existence of the binary toxin, CDT, has demonstrated an augmentation in inflammasome activation via TLR2-mediated priming [45,133]. The process involves the coordinated actions of Mitogen-Activated Protein Kinases (MAPKs), which in turn activate the Nuclear Factor κB (NF-κB) [133]. MAPKs are triggered, initiating a phosphorylation cascade that leads to the degradation of IκB, enabling the release and translocation of NF-κB into the nucleus for the transcriptional regulation of inflammatory genes [134]. This collaborative interplay among the inflammasome, NF-κB, and MAPKs forms a dynamic and orchestrated response, pivotal for the host’s early defense against *C. difficile* infection.

In severe cases, pseudomembrane plaques are visible on the colonic mucosa around day 3 and 4 p.i., consisting of inflammatory cells, fibrin, and mucus [135]. However, it is essential to recognize that the mouse model of CDI is typically acute, encompassing a brief one-week timeframe [54,80]. After the disease reaches its peak around day 2 and 3 p.i., a resolutive phase takes hold within the intestinal lamina propria around 5 days postinfection [79]. During this phase, we observe a diminishing inflammatory response, with increased epithelial cell proliferation and mucus production, and the gradual relief of clinical symptoms, including body weight loss and diarrhea [79,136,137]. Significant changes include a decline in neutrophil infiltration in the tissue, a decrease in proinflammatory cytokine levels, and an increase in IL-10 and IL-22 production [138,139,140,141]. Moreover, there is a notable increase in the prevalence of group 3 innate lymphoid cells (ILC3) and regulatory T cells (Treg cells) within the lamina propria, with these alterations becoming particularly pronounced after 5–6 days of infection [128,142]. IL-22-productin ILC3 play a crucial role in CDI, reinforcing the gut barrier and promoting tissue repair [121,128]. Treg cells have a dual role, modulating immune responses to prevent excessive inflammation, while potentially hampering the efficient clearance of *C. difficile* [142,143].

In some instances, the immune response in mice is effective in clearing the infection, and they recover from CDI. However, chronic colonization can occur in some cases (around 20% of cases), with *C. difficile* persisting in the gut, potentially leading to recurrent episodes of infection [144,145]. Furthermore, following a recovery period and the discontinuation of antibiotics, mirroring the clinical scenario in which patients are susceptible to recurrent CDI, mice can be subjected to a rechallenge with *C. difficile*, whether from the same or a different strain [54]. The rechallenge initiates a recurrent phase in mice that were previously infected, enabling the evaluation of the host’s immune response, the maturation of protective immunity, and the effective assessment of various interventions for preventing relapse. Although T cells and B cells may not play a direct role in resolving the acute phase of *C. difficile* infection in mice [128,141], clinical data suggest that adaptive immune responses can confer protective effects. In this context, B cells are instrumental in the production of specific antibodies, including IgG and IgA, which can neutralize *C. difficile* toxins and limit their damage to intestinal epithelial cells [21,146,147,148]. Furthermore, CD4+ and CD8+ T cells play pivotal roles, orchestrating the immune response by aiding in antibody production, stimulating other immune cells, and directly targeting infected host cells [149,150,151]. Importantly, the development of immunological memory within the adaptive immune system ensures a more rapid and effective response upon re-exposure to *C. difficile* [152,153].

In the murine model, CDI manifests as clinical symptoms similar to those seen in humans. The severity of symptoms can vary depending on factors such as the *C. difficile* strain used, the antibiotic regimen, and the specific study conditions. In general, symptoms include severe diarrhea, often watery in consistency, which reflects the damaging effects of *C. difficile* toxins on intestinal epithelial cells [4]. In mice, CDI leads to significant weight loss, typically ranging from 10% to 20% of their initial body weight [80], serving as a quantifiable marker of infection severity and its overall impact on host responses. Reduced activity, lethargy, and altered grooming behavior are also common behavioral manifestations after infection [107]. Additional clinical signs, such as a hunched posture and ruffled fur, further highlight the mice’s discomfort [77]. These signs and symptoms are commonly quantified using a standardized clinical scoring system, frequently employed in research and experimentation (Table 1) [77].

Furthermore, colonic inflammation characterized by neutrophil infiltration and tissue damage, and pseudomembranous colitis featuring visible plaque on the colonic mucosa, serve as critical histopathological features [54,123]. Additional parameters, including a decrease in goblet cell frequency and mucus reduction, also serve as indicators of disease progression and severity [154,155]. The assessment of epithelial permeability, exemplified by tests involving the ingestion of sugar molecules followed by the analysis of urine or blood samples, as well as the quantification of translocated bacteria in various peripheral organs (e.g., liver, spleen, kidney, etc.), can serve as valuable indicators of compromised epithelial barrier function following infection [120]. Finally, examination of the immune response within the intestinal tissue postinfection, conducted through techniques like flow cytometry, immunofluorescence on preserved tissue sections, or the quantification of cytokines and gene expression, serves as a valuable means to monitor the progression of CDI in murine models [6].

## 6. Conclusions

Over time, *C. difficile* is increasingly developing resistance to antibiotics and generating virulent strains, posing challenges for healthcare providers and researchers alike. Current research on CDI covers a wide range of crucial areas, including the exploration of the gut microbiome’s role, investigating alternatives to antibiotics such as fecal microbiota transplantation (FMT), and finding strategies for the prevention and management of CDI. Mouse models have played a crucial role in the understanding of this field, offering insights into pathogenesis, host responses, and potential interventions. Researchers have employed various mouse models in the field, including genetically modified and germ-free mice. Specifically, the antibiotic-induced mouse model stands as a well-established tool for studying CDI, aiding in the comprehension of host–pathogen interactions. While it does have many benefits, the model may not fully replicate the complexities of human CDI, as it possesses gut microbiota that differ from that of humans. Overall, various protocols for utilizing mice in experimental CDI have been crucial for advancing our understanding of the disease and facilitating the development of approaches that may potentially be applied for managing the disease.

## Figures and Tables

**Table 1:** Clinical score for evaluating the murine *C. difficile* infection.

Category	Score *			
	0	1	2	3
**Activity**	Normal	Alert/Slow moving	Lethargic/Shaky	Inactive unless prodded
**Posture**	Normal	Back slanted	Hunched	Hunched/Nose down
**Pelage**	Normal	Piloerection	Rough skin	Very ruffled Puff/Ungroomed
**Diarrhea**	Normal	Soft stool/Discolored (yellowish)	Wet stained tail/mucous +/− blood	Liquid or absence of stool (ileus)
**Eyes/Nose**	Normal	Squinted ^1^/_2_ closed	Squinted/Discharge	Closed/Discharge

* Clinical score = sum of all parameter scores. Total possible score is 15 (death).

## Data Availability

This review article does not involve the generation of new data, as it relies solely on the analysis and synthesis of information from existing literature. All referenced sources are appropriately cited within the manuscript.
